# Comparison of Machine Learning Algorithms Fed with Mobility-Related and Baropodometric Measurements to Identify Temporomandibular Disorders

**DOI:** 10.3390/s24113646

**Published:** 2024-06-04

**Authors:** Juri Taborri, Luca Molinaro, Luca Russo, Valerio Palmerini, Alin Larion, Stefano Rossi

**Affiliations:** 1 Department of Economics, Engineering, Society and Business Organization (DEIM), University of Tuscia, 01100 Viterbo, Italy; luca.molinaro@unitus.it (L.M.); stefano.rossi@unitus.it (S.R.); 2 Department of Human Sciences, Università Telematica Degli Studi IUL, 50122 Florence, Italy; info@dottlucarusso.com; 3 Department of Rehabilitation, Faculty of Medicine, University of Ostrava, 00183 Rome, Italy; valerio.palmerini@gmail.com; 4 Faculty of Physical Education and Sport, Ovidius University of Constanta, 900029 Constanta, Romania; alinlarion@yahoo.com

**Keywords:** machine learning, temporomandibular disorder, inertial sensors, pressure platform, clinical assessment

## Abstract

Temporomandibular disorders (TMDs) refer to a group of conditions that affect the temporomandibular joint, causing pain and dysfunction in the jaw joint and related muscles. The diagnosis of TMDs typically involves clinical assessment through operator-based physical examination, a self-reported questionnaire and imaging studies. To objectivize the measurement of TMD, this study aims at investigating the feasibility of using machine-learning algorithms fed with data gathered from low-cost and portable instruments to identify the presence of TMD in adult subjects. Through this aim, the experimental protocol involved fifty participants, equally distributed between TMD and healthy subjects, acting as a control group. The diagnosis of TMD was performed by a skilled operator through the typical clinical scale. Participants underwent a baropodometric analysis by using a pressure matrix and the evaluation of the cervical mobility through inertial sensors. Nine machine-learning algorithms belonging to support vector machine, k-nearest neighbours and decision tree algorithms were compared. The k-nearest neighbours algorithm based on cosine distance was found to be the best performing, achieving performances of 0.94, 0.94 and 0.08 for the accuracy, F1-score and G-index, respectively. These findings open the possibility of using such methodology to support the diagnosis of TMDs in clinical environments.

## 1. Introduction

Temporomandibular disorders (TMDs) include a group of clinical signs and symptoms in the temporomandibular joints, masticatory system and related structures [1]. TMD affects 5–12% of the population [2]; in addition, it has been confirmed that more women suffer from TMDs than men, with a prevalence of 6.3%, compared to 2.8% [3]. Symptoms of TMD include pain and/or changes to temporomandibular joint (TMJ) functions; specifically, pain in the jaw joint and/or masticatory muscles, limited mobility and joint crepitus are the three most representative symptoms [4]. The causes of TMD include structural factors (e.g., skeletal, neural and muscular), functional factors (e.g., posture and lifestyle), and psychological factors (e.g., stress), or any combination of them [1].

The evaluation of TMD typically involves a multifaceted approach that integrates various clinical assessments, imaging techniques and subjective patient-reported measures [5]. Clinical examination typically includes: (i) the assessment of jaw mobility and function; (ii) palpation of the TMJ and surrounding muscles to identify tenderness, swelling or muscle spasms; (iii) evaluation of occlusion; and (iv) assessment of associated symptoms. Imaging modalities may include X-rays, magnetic resonance imaging (MRI) and computed tomography (CT), providing valuable information about the TMJ and surrounding structures [6]. However, the risks associated with radiological exposure and the costs of these types of assessments cannot be overlooked, and the availability of specialized clinical laboratories, and the need to be able to monitor rehabilitation treatments also need to be considered [7]. In addition to an instrumented-based approach, patient-reported outcome measures can provide valuable information about the impact of TMD on daily functioning, quality of life and psychological factors such as anxiety or depression [2]. Common questionnaires include the Oral Health Impact Profile (OHIP), Jaw Function Limitation Scale (JFLS) and TMD-specific questionnaires like the Research Diagnostic Criteria for Temporomandibular Disorders (RDC/TMD). By combining these various assessment methods, clinicians can obtain a comprehensive understanding of the patient’s TMDs presentation, allowing for accurate diagnosis and appropriate treatment planning [8]. These rating scales, despite being considered the gold standard for more than 25 years, are continually placed under review for their validity and reliability [9] and questioned among clinical operators [10].

Aiming to improve the quality of the diagnosis and monitoring, much attention in recent years has been paid to the postural assessment of people affected by TMDs. Many studies have focused on the evaluation of posture and mobility of the cervical spine, whereas others have focused on the quality of balance management and ground support. Walczyńska-Dragon et al. evaluated the influence of TMD therapy on cervical spine range of movement (ROM), analyzed by an ultrasound device, and reduction in spinal pain [11]. After 3 months of therapy the results showed a significant improvement in TMJ function and cervical spine ROM, and a reduction in spinal pain demonstrating a significant association between TMD treatment and reduction in cervical spine pain, as far as improvement in cervical spine mobility. Grondin et al. used an inclinometer to evaluate cervical ROM measures in twenty asymptomatic subjects compared with 37 subjects with pain attributed to TMDs [12]. The researchers highlighted significant differences between groups indicating that subjects with TMDs had signs of upper cervical spine ROM impairment. Regarding body posture, Nota et al. have highlighted that there is a significant difference in the postural stability of the body between subjects suffering from myogenic TMD and healthy controls [13]. In particular, the postural parameters of the oscillation area and of the oscillation speed were increased when gathered from participants affected by TMDs. Souza et al. evaluated body posture through photogrammetry and the distribution of plantar pressure, with a pressure platform, at physiologic rest of the mandible and during maximal intercuspal positions in subjects with and without TMDs [14]. They found a more pronounced cervical distance, valgus of the right calcaneus and lower pelvic tilt in the photogrammetric analysis and a higher rearfoot and lower forefoot distribution for the TMD subjects, while no differences were verified in maximal intercuspal position in the between-group analysis and between the two mandibular positions in the within-group analysis. In contrast, Scharnweber et al. did not find evidence of correlations between the parameters provided by a pressure platform during a static and dynamic examination in subjects with different dental occlusions, concluding that postural control and plantar pressure distribution are independent of each other [15]. Despite the plethora of studies published on the topic, the correlation between postural assessments and TMDs remains unclear [16,17,18].

Considering these aspects, as well as the notable advances in artificial intelligence (AI), the application of machine learning algorithms appears to be a promising approach for diagnosis in clinics, such as for TMDs [19], and in sports [20,21]. In particular, AI algorithms can analyse medical imaging data gathered by MRI, CT scans and X-rays to identify anatomical abnormalities, joint degeneration or structural irregularities indicative of TMDs. Machine learning models, particularly convolutional neural networks (CNNs), have been demonstrated to excel in image recognition tasks and can assist in automating the detection and classification of TMD-related pathologies. Reda et al. [22] proposed an architecture based on commercially available cognitive computing services, trained using scientific documents and interviews with experienced professionals regarding the diagnosis of TMDs. They give preliminary proof of the feasibility of implementing an AI-based system to support untrained dentists in the recognition of TMDs. Furthermore, the work conducted by Lee et al. aimed to identify contributing biological and psychosocial factors and their relative importance as risk factors for the development of TMDs using artificial intelligence methodologies on a nationwide sample of 4744 participants [23]. They used six artificial intelligence approaches, SVM, artificial neural network (ANN), random forest, naïve Bayes, decision tree and logistic regression for identifying the factors associated with TMDs. Their results highlight the importance of obesity, general health, stress, socioeconomic status and working conditions in the management of TMDs.

Although the promising usefulness of artificial intelligence in providing automatic information for the diagnosis and classification of TMDs is evident, the already published papers, to the best of the authors’ knowledge, are focused on data gathered from high-cost and not-portable technologies, such as MRI and CT, or not associated with physical functions. Thus, the aim of the present study is to propose a sensor system based on low-cost and portable technologies, such as inertial sensors and a baropodometric platform, in order to gather data to feed machine-learning algorithms for the identification of TMDs. From this perspective, we performed a comparative analysis among the most widely used machine-learning algorithms fed with data related to baropodometric analysis and cervical mobility to define the best performing one.

The findings of the study can represent a starting point to foster the use of portable sensor systems to objectivize the identification of TMDs in clinical settings.

## 2. Materials and Methods

### 2.1. Participants

Fifty subjects (20 males and 30 females) were involved in the study. They were divided into two homogeneous groups of 10 males and 15 females: a group of healthy subjects acting as a control group (CG) (height = 172 ± 8 cm, body mass = 68 ± 13 kg, age = 31 ± 6 years) and a group with TMD (TMD) (height = 172 ± 10 cm, body mass = 69 ± 14 kg, age = 32 ± 7 years). The division into the two groups was carried out through a clinical examination performed by an experienced operator in combination with two questionnaires on anxiety and depression. For recruitment the exclusion criteria were the following: (a) no orthodontic treatment in progress; (b) having undergone orthognathic surgery; (c) diagnosis of other painful orofacial conditions; (d) primary disorder of the cervical spine such as herniated disc or significant degenerative changes in the spine; (e) systemic disease that could compromise the mobility of the spine such as spondylitis; (f) reported injuries or accidents involving the head or vertebral column. All participants were initially informed about the purpose of the study and asked to sign a written consent. The experimental procedures were in accordance with the principles set out in the Declaration of Helsinki. The study has been approved by the Institutional Review Board of the Ovidius University of Constanta (Romania) with the protocol n. 45, dated 23 January 2024.

### 2.2. Experimental Protocol and Setup

The experimental protocol was composed of two distinct evaluations: the first consisted in the clinical evaluation of the subjects, whereas in the second part the instrumental evaluation was carried out. Both assessments were carried out on the same day and the complete protocol lasted approximately 30 min per participant.

#### 2.2.1. Clinical Assessment

The clinical evaluation protocol was structured in two parts. The first part focused on the physical aspects of the patient and was based on the recent review of Malgorzata et al. on the *Diagnostic of Temporomandibular Disorders and Other Facial Pain Conditions* [24]. The protocol was carried out by a physiotherapist with advanced training in the manual therapy of the TMJ and cervical spine. The second part consisted of the administration of two questionnaires: the Patient Health Quality 9-item (PHQ-9) and the Generalized Anxiety Disorder 7-item (GAD-7). These questionnaires are part of Annex II of the Diagnostic Criteria for Temporomandibular Disorder (DCTMD) [25], which is the standard tool used to assess TMD, considering both the physical and psychosocial aspects of the patient.

The first part of the clinical evaluation involved 5 steps:

##### Mouth Opening (MO)

**Figure 1:**
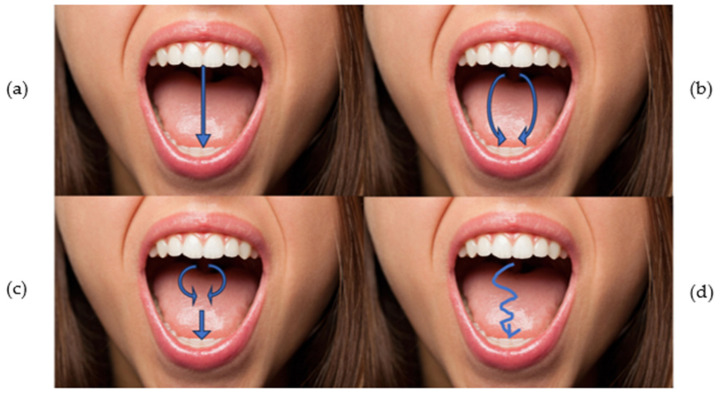
(**a**) Straight opening; (**b**) lateral deviation; (**c**) corrected deviation and (**d**) other opening. Arrows indicate the type of movement.

The operator asked the subject to open and close the mouth three times, evaluating only the opening phase [24] (Figure 1). The movement was recorded with a smartphone so that it could be reviewed in post-processing analysis, if needed. If the participant had several opening modalities, he/she was asked to repeat the three openings and was then classified according to the following criteria:

- Straight (S). No deviation is observed.
- Lateral deviation to the right (RDEV) or to the left (LDEV). For deviations that are observed in the maximum aperture on one side only, the operator determined which side of the face the deviation is directed to and noted it.
- Corrected deviation (CDEV). The patient has a slight deviation, to the right or to the left, which is corrected either at the midline or when the maximum unguided opening is reached.
- Other (O). Participant showed a discontinuous or different opening than those listed.

##### Opening Width (OW)

**Figure 2:**
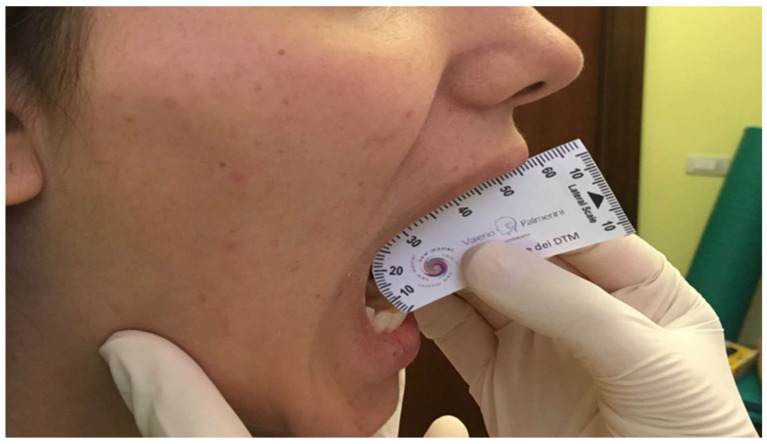
Measuring the width of the opening.

The operator asked the participant to place the lower jaw in a comfortable position and open the mouth as wide as possible until no pain is felt [24]. At this point, the operator positioned the ruler vertically by measuring the distance between the edge of the upright upper incisor and the edge of the corresponding lower incisor as shown in Figure 2.

If the opening was less than 30 mm, the measurement was repeated twice. If the aperture was less than 30 mm the second time, it was recorded.

##### Joint Noises (JN)

The operator identified the presence and type of noise when the subject was opening the mouth. The operator placed the left index finger on the right TMJ and the right index finger on the left TMJ in front of the tragus [24]. He/she then asked the participant to slowly open his/her mouth as much as possible, even in the presence of pain, and then to close his mouth so as to bring the teeth into maximum intercuspation. The movement was repeated three times. A stethoscope was used during the procedure, even though it is not mandatory for the application of the DCTMD procedure. The operator noted the outcome of the examination according to three criteria:

0 = No noise.

1 = Click. A defined noise, of very short duration, with a distinct beginning and end, which is usually felt as a click. This noise was only noted if it could be reproduced in two of the three opening and closing movements.

2 = Crepitus. Continuous noise, which lasts longer than the click. Present for a longer period during the opening and closing movement. Crackling can manifest itself as continuous, overlapping noises. This noise is not muffled and can be described as the sound of bone rubbing against bone.

##### Muscle Palpation (MP)

**Figure 3:**
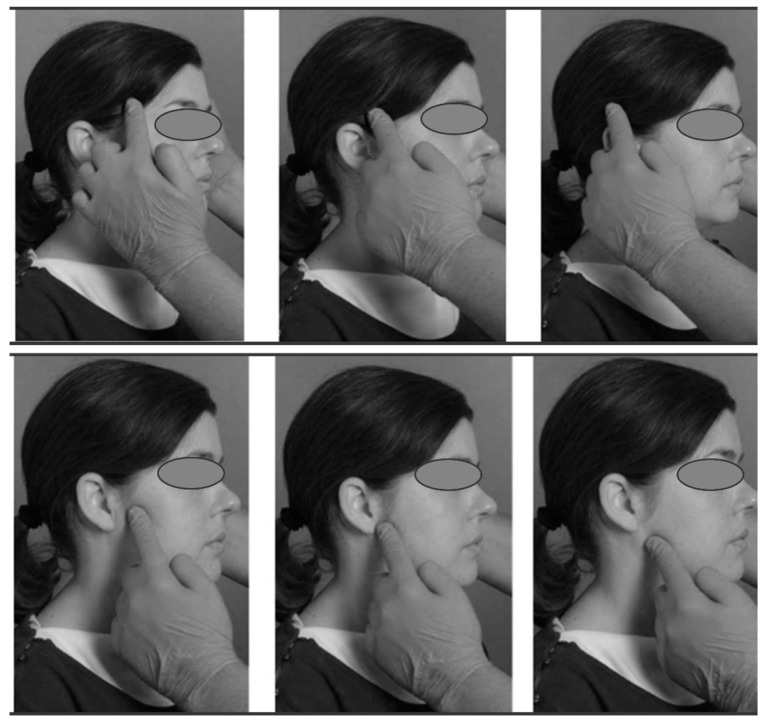
Muscle palpation: the upper images demonstrate the palpation of the temporal muscle and the lower images demonstrate palpation of the masseter muscle.

Muscle palpation was performed by pressing with the tip of the index finger on the site of interest while with the other hand the operator held the subject’s head on the side opposite to the one examined. The lower jaw had to be in a resting position, with the teeth exposed. To locate the muscle site well, the operator asked the participant to clench his/her teeth slightly and then relax the muscles [24]. Since areas of maximum pain are variable from patient to patient, different areas of the muscle were palpated to determine if pain was present as shown in Figure 3. The muscles evaluated were the temporalis muscle and the masseter muscle.

During palpation, the operator asked if pain or pressure was felt on certain parts of the head or face. The goal was to stimulate, during palpation, the familial pain, that is, a pain that the subject knows well and can recognize as his own. Each muscle palpation lasted 5 s for each point.

##### Joint Palpation (JP)

**Figure 4:**
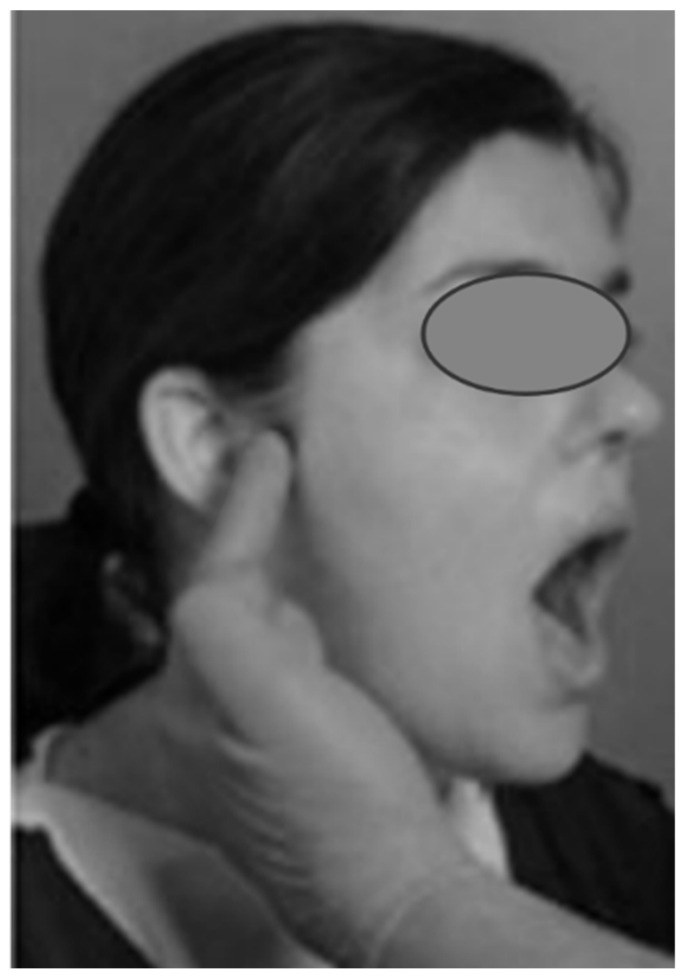
Joint palpation.

For joint palpation, the operator placed the index finger immediately in front of the tragus and above the temporomandibular joint, applying a slight pressure as shown in Figure 4. At the same time, with the other hand on the opposite side holding the head, the subject was asked to open and close the mouth [24]. During palpation, the operator asked if pain or pressure was felt on the joint in the back or front.

##### PHQ-9 and GAD-7

The Patient Health Questionnaire 9-item Depression Scale (PHQ-9) and the 7-item Generalized Anxiety Disorder Scale (GAD-7) are among the best-validated and most commonly used measures of depression and anxiety, which represent two psychological variables affecting the occurrence of TMDs [26]. They have been used in hundreds of research studies, incorporated into numerous clinical practice guidelines, and adopted by a variety of medical practice and mental health settings.

The PHQ-9 consists of 9 items that represent symptoms for DSM 5 major depressive disorder. Respondents are asked how much each symptom has bothered them over the past 2 weeks.

The GAD-7 has 7 items. Although originally developed as a measure to detect generalized anxiety disorder, the operational features of GAD-7 are almost as valid for other anxiety disorders common in clinical practice such as panic disorder, social anxiety disorder and post-traumatic stress disorder. The PHQ-9 and GAD-7 have strong internal and test–retest reliability, as well as construct validity and factor structure [27].

#### 2.2.2. Instrumental Assessment

##### Baropodometric Analysis

**Figure 5:**
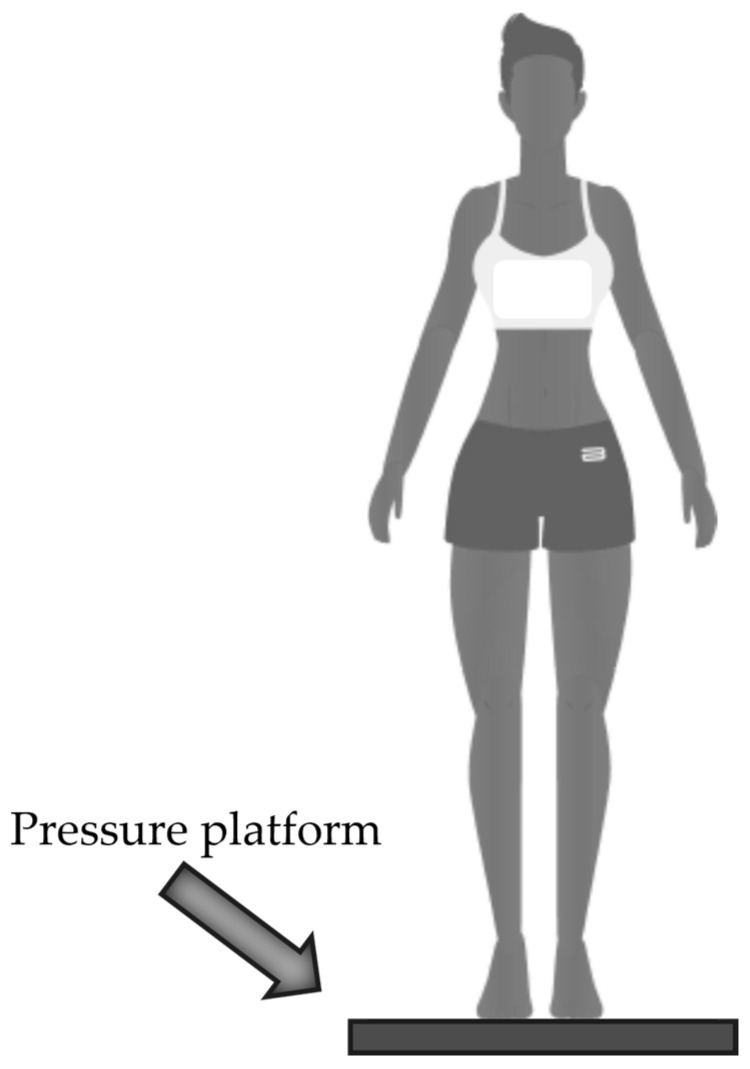
Baropodometric setup.

The freeMed BASIC 40 × 40 platform (Sensor Medica, Guidonia Montecelio, Italy) was used for the acquisition of baropodometric data. The platform is composed of 2.400 resistive 24 k coated gold sensors arranged according to 40 rows and 40 columns for a total area of 398 × 318 mm^2^. The full scale of the sensors is 150 N/cm^2^ with an acquisition frequency up to 400 Hz and the sensitive area of each sensor is 1 × 1 cm^2^. The matrix is covered with a conductive synthetic rubber material in socaprene and is connected to the PC via USB cable and interfaces with the freeSTEP 2.0 software (Sensor Medica, Guidonia Montecelio, Italy, version 2.00.010). The pressure platform is widely used in both clinical and sports applications [28,29,30] and its reliability and repeatability has already been analyzed by the same authors [31]. The experimental protocol consisted in a static task, where participants were asked to take off their footwear and step on the platform with a self-selected position of the feet. Once the subject was positioned on the platform, with relaxed arms, the subject was asked to look forward towards a target, which was placed at a distance of 5 m, in order to stabilize the posture (Figure 5). The duration of the static test was 5 s and was repeated three times with a 30 s break between trials. Once the acquisition was completed, the participant stepped off the platform and then repositioned him/herself on it. The test was repeated three times. Data were collected at a 50 Hz.

##### Cervical ROM Analysis

**Figure 6:**
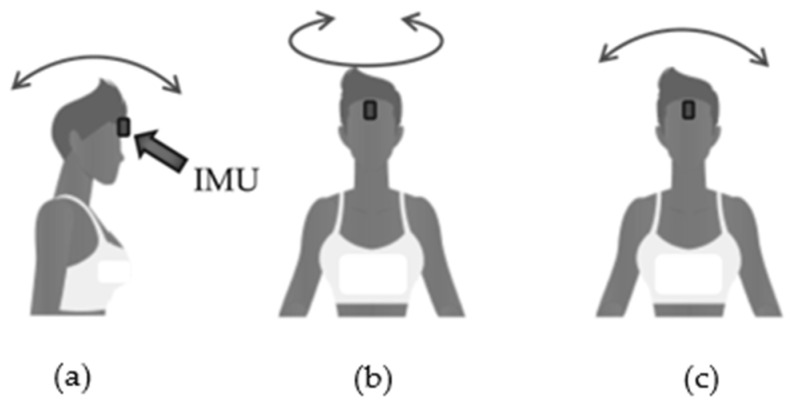
Cervical mobility with Moover sensor. (**a**) Flexion–extension of the head; (**b**) head rotation and (**c**) head inclination. Arrows indicate the relative rotation.

Cervical mobility tests were acquired with the Moover sensor (Sensor Medica, Guidonia Montecelio, Italy). The Moover device consists of an inertial measurement unit (IMU), with dimensions of 65 mm × 45 mm × 18 mm and a mass of 28 g, which contains a triaxial accelerometer, gyroscope and magnetometer with full scales of ±16 g, ±2000 °/s and ±4800 µT, respectively. The device is equipped with an internal 32-bit processor with a floating point unit that processes the data and sends the results via Bluetooth 4.0 connection to the PC to be stored and analyzed using the freeSTEP 2.0 software (Sensor Medica, Guidonia Montecelio, Italy, version 2.00.010). The sampling frequency was set at 200 Hz. The validity of the device for the evaluation of cervical ROMs was analyzed in a recent article [32]. The device was fixed on the head, in particular on the forehead, using a special semi-elastic belt equipped with specific device support to avoid relative movements during the execution of the task. The elastic waistband ensures subject comfort and reduces motion artifacts during task performance. The cervical mobility assessment was carried out standing with arms relaxed at the sides; participants were asked to fix a target placed 5 m away in front to standardize the starting position and to acquire a static phase for the inertial sensor. At this point, three distinct movements were carried out as shown in Figure 6: (a) flexion–extension of the head; (b) rotation of the head towards the two sides and (c) inclination of the head to both sides. Each test included a total of eight movements with four repetitions performed for both sides. Participants were free to start towards whichever side they preferred. An expert operator paid attention to any compensatory movements of the body, in which case the participant was asked to repeat the test.

### 2.3. Data Analysis and Feature Extraction

#### 2.3.1. Clinical Assessment

The clinical assessment was conducted in order to categorize each subject and to have the label for the supervised application of machine-learning algorithms. Each item of the DCTMD protocol was analyzed independently.

Concerning the mouth opening (MO), a score of 0 or 1 was assigned, following the rules reported in Table 1. Thus, each subject could obtain a minimum score equal to 0 and a maximum score equal to 4.

Moving to the analysis of opening width (OW), the index reported is the millimeters measured through the proper graduated ruler, whereas the joint noise (JN) task was analyzed according to the values reported in Table 2, with the score ranging from 0 to 2.

Considering the muscle and joint palpation, MP and JP, respectively, the operator could assign 0 or 1 for each specific muscle/joint, where 0 and 1 indicate the absence and presence of the pain, respectively, as in Table 3. Thus, each subject could obtain a minimum score equal to 0 and a maximum score equal to 3.

##### PHQ-9 and GAD-7

Finally, the analysis of the two administered questionnaires, the PHQ-9 and GAD-7 questionnaires, followed the indications reported in Table 4 and Table 5, respectively.

The PHQ-9 can be assessed as a continuous variable from 0 to 27 (with higher scores representing more severe depression) or categorically using a diagnostic algorithm for major depression or other depressive disorders [27]. GAD-7 presents identical responses to the PHQ-9 and therefore can be assessed as a continuous variable from 0 to 21 (with higher scores representing more severe anxiety). The most commonly used cut-off value on both PHQ-9 and GAD-7 for screening for depressive and anxiety disorders, respectively, is 10 or higher.

By combining the results of the clinical tests through the application of the diagnostic decision trees reported in [33], it is possible to diagnose the presence of TMD. The decision tree has been applied by the same skilled operator, who performed the clinical assessment.

#### 2.3.2. Instrumental Assessment

##### Baropodometric Analysis

**Figure 7:**
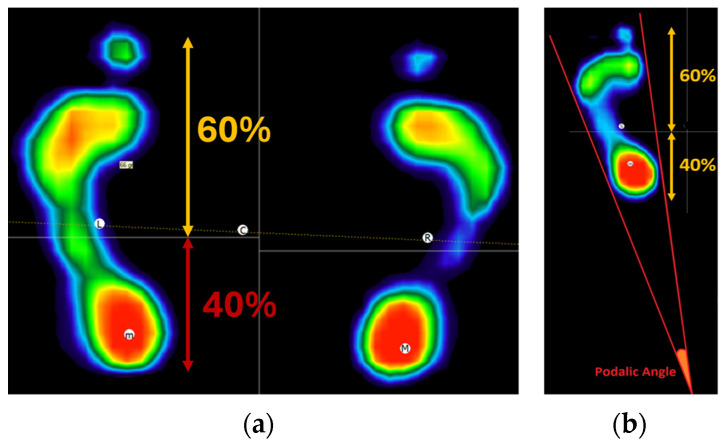
(**a**) Representation of static test and the division of the forefoot and rearfoot. (**b**) Calculation of the podalic angle. Color map indicates the level of pressure with the lowest related to the blue and the highest to the red.

After placing the subject on the platform, the software automatically calibrates itself based on the subject’s weight in order to avoid the saturation of the platform amplifiers. Once the acquisition phase has been completed, the software automatically distinguishes between the right and left feet, dividing their footprint into forefoot and rearfoot regions based on pressure distribution, with 60% allocated to the forefoot and 40% to the rearfoot (Figure 7a). The analysis focused on the following parameters:

- The podalic angle (PA) is the angle formed by the intersection of the two lateral tangents of the footprint (Figure 7b).
- Forefoot and rearfoot load percentage (FL and RL, respectively) computed as the load distribution on the forefoot and rearfoot estimated from the subject weight.
- Total load percentage between left and right foot (TL), indicating the percentage of the total weight distributed between the right and left side.

Each parameter was calculated for both the left and right footprint and the average value was calculated across the three repetitions, individually per each subject.

##### Cervical ROM Analysis

Regarding cervical mobility tests, the freeSTEP 2.0 software automatically realigned the Moover axes with the absolute reference system using data gathered during the initial static phase. The sensor’s orientation was determined by merging linear accelerations and angular velocities using a fusion algorithm based on the Mahony filter [34]. For each test, the first and last values of the 8 rotation movements were discarded. The average value, indicated with *θ*, was then calculated, considering the following parameters, all expressed in degree:

- Flexion [*θ_FLEX_*]: the value of the maximum angle reached in flexion.
- Extension [*θ_EXT_*]: the value of the maximum angle reached in extension.
- Total flexion–extension [*θ_FLEX+EXT_*]: the sum of the two angles of flexion and extension.
- Right rotation [*θ_R_ROT_*]: the value of the maximum angle reached in rotation to the right.
- Left rotation [*θ_L_ROT_*]: the value of the maximum angle reached in rotation to the left.
- Total rotation [*θ_TOT_ROT_*]: the sum of the two right and left rotation angles.
- Right inclination [*θ_R_INC_*]: the value of the maximum angle reached in the inclination to the right.
- Left inclination [*θ_L_INC_*]: the value of the maximum angle reached when tilting to the left.
- Total inclination [*θ_TOT_INC_*]: the sum of the two right and left inclination angles.

### 2.4. Machine-Learning Algorithms

To understand if a supervised machine-learning-based approach can help to objectively assess the occurrence of TMD through the analysis of physical parameters associated with cervical mobility and baropodometric analyses, we conducted an investigation into two distinct categories of supervised machine learning algorithms, geometric and binary, as they are among the most commonly used algorithms for motion recognition [35]. Within the geometric classifiers category, we chose to focus on the support vector machine (SVM) and the k-nearest neighbor (kNN), whereas the decision tree (DT) was selected from the binary algorithms.

SVMs are geometric supervised machine-learning algorithms that aim to identify the hyperplane that best separates features belonging to different classes [36]. The choice of kernel function, which linearizes the feature space, is crucial before initiating the classification process. Specifically, we tested three SVMs: linear (l-SVM), quadratic (q-SVM) and cubic (c-SVM).

kNNs are geometric supervised machine learning algorithms that make classification decisions by identifying the most frequent class among the k-nearest neighbors while maximizing the distance from other classes [37]. The distance computation equation is a key parameter to select. We examined three kNN variants: fine kNN (f-kNN), employing Euclidean distance with one neighbor; cosine kNN (c-kNN), utilizing cosine distance with ten neighbors; and weighted kNN (w-kNN), employing weighted Euclidean distance with ten neighbors based on a squared inverse approach.

Finally, DTs are binary supervised machine learning algorithms that predict the most probable class by constructing a series of nodes, wherein classification occurs through specific splitting criteria. For this study, the split criterion was based on the Gini index. The maximum number of splits is a critical parameter to choose before classification. We tested three DT variations: coarse (c-DT), medium (m-DT) and complex DT (cx-DT), with maximum split numbers set at 4, 20 and 100, respectively [21].

In summary, nine machine-learning algorithms were tested and compared. Each algorithm was fed by using the synthetic indices extracted from the baropodometric analysis, i.e., eight features, four per each foot, and the ones associated with the cervical mobility, for a total of nine features. To evaluate which was the best solution for the feature selection, three datasets of features were constructed by considering the following: (i) only the features obtained from the baropodometric test, condition B; (ii) only the features gathered from the cervical mobility analysis, condition M; and (iii) all features together, for a total of 17 features, condition MB.

The performance of the tested machine-learning algorithms was evaluated by using a 10-fold cross-validation. Consequently, we divided the datasets into 40 subjects for training and the remaining ten for validation. Finally, the algorithms’ performance was obtained by averaging across all folds. The supervised approach has been guaranteed by using the output of the clinical scales to label the data in the TMD group and CG.

#### Performance Metrics

The classes “TMD”, which includes subjects manifesting temporomandibular disorder, and “CG”, which includes subjects belonging to the control group, that were predicted by the aforementioned algorithms were compared to the reference values obtained through the analysis of the clinical assessment. For each classifier, a two-by-two confusion matrix was generated. Subsequently, algorithms performances were evaluated in terms of accuracy, F1-Score and goodness index. Accuracy (A) was calculated as the ratio of correctly predicted risk classes to the total predictions using the following Equation (1):

(1) $$A=\frac{\mathrm{TP}+\mathrm{TN}}{\mathrm{TP}+\mathrm{TN}+\mathrm{FP}+\mathrm{FN}}$$

where TP, TN, FP and FN denote true positive, true negative, false positive and false negative, respectively. True positive and true negative were obtained when the algorithm correctly classified subjects as “TMD” or “CG” (R), respectively, consistent with the clinical assessment. A value close to 1 indicates a perfect classifier, with 0.80 typically chosen as the threshold for an optimal classifier.

The F1-score, a harmonic average of recall (Re) and precision (P), where recall is the ratio of true positive to the sum of true positive and false positive, and precision is the ratio of true positive to the sum of true positive and false positive, was computed as follows (Equation (2)):

(2) F1−score=2·(Re·P)(Re+P)

Similar to accuracy, the F1-score ranges from zero to one, with a value close to 1 indicating a perfect classifier and a threshold of 0.80 indicating an optimal classifier. Additionally, the goodness index (G), representing the Euclidean distance in the receiver operating characteristic space between the tested classifier and the perfect one, was computed as follows (Equation (3)):

(3) $$G=\sqrt{{\left(1-\mathrm{TP}\right)}^{2}+{\left(1-\mathrm{TN}\right)}^{2}}$$

G ranges from 0 to $\sqrt{2}$ with the following goodness ranges: (i) optimum when G ≤ 0.25; (ii) good when 0.25 < G ≤ 0.70; (iii) random if G = 0.70; and (iv) bad if G > 0.70 [38].

## 3. Results

The results of the clinical assessments led to the identification of twenty-five participants belonging to the TMD class and the remaining twenty-five to the CG. Specifically, the values gathered from the clinical assessment are reported in Table 6.

It is clear that the TMD group was associated with a lower averaged value of mouth opening, as well as with higher scores for both administered questionnaires in comparison to participants belonging to the CG. In addition, all of the participants belonging to the TMD group manifested deviation during mouth opening, noise and pain at both the muscle and joint level.

**Figure 8:**
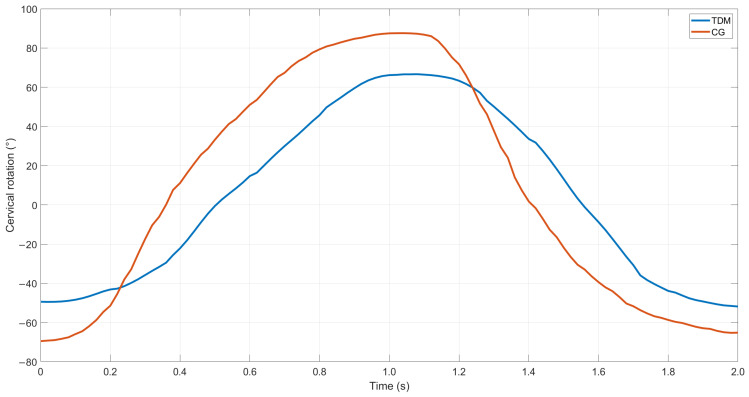
Example of left cervical rotation, expressed in degree, of a single repetition for CG (red line) and TMD group (blue line).

**Figure 9:**
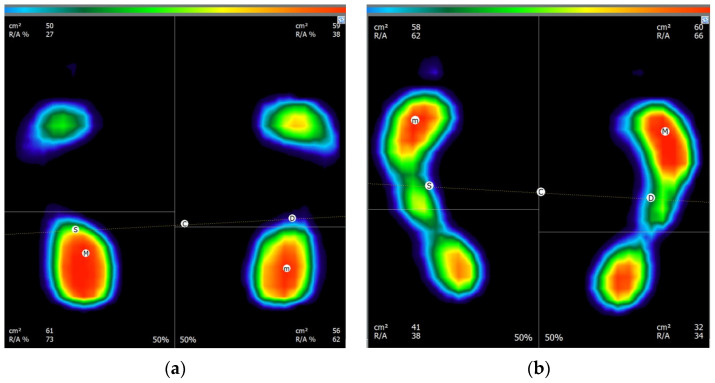
Example of baropodometric result for CG (**a**) and TMD group (**b**). Color scale represents the amount of load, with greater load associated with red color.

Moving to the instrumented analysis, as an example, the waveform associated with the cervical rotation belonging to either TMD group and CG, as well as the graph of the baropodometric analysis, are reported in Figure 8 and Figure 9, respectively.

From the above reported example, it is possible to observe how the subject belonging to the TMD group was characterized by a lower value in the cervical rotation, both for the right and left directions, i.e., the positive and negative values, respectively, in comparison to the CG behavior. Similarly, the two subjects were characterized by different behavior on the baropodometric analysis in terms of load distribution. Specifically, a greater amount of load was distributed on the forefoot for the TMD group, whereas a greater load on the rearfoot was observed for the CG. For the sake of clarity, Table 7 reports the mean values and standard deviations for all of the computed features for both groups.

Analyzing the values of the indices gathered from the baropodometric analysis, it is possible to affirm that CG participants tend, in general, to unload the weight more on the rearfoot, whereas the opposite behavior can be observed for TMD. Considering the PA, the TMD group was found to be characterized, on average, by a lower value in the podalic angle. Finally, no qualitative differences can be appreciated for the distribution of the weight between the left and right foot, considering the two groups.

By moving to the cervical mobility, it is evident that the TMD group is always associated, on average, with a lower mobility for all of the tested movements, with the greatest differences observed for flexion/extension and left and right rotation.

Table 8 shows the results in terms of accuracy, F1-score and goodness obtained for all of the tested classifiers. By analyzing the results reported in the above table, it was revealed that the best classifier was the one based on the c-kNN trained with the dataset composed of features gathered from both mobility and baropodometric analyses. In particular, the classifier is associated with an accuracy, F1-score and G-index equal to 0.94, 0.94 and 0.08, respectively. All of these three reported values are in line with the threshold for defining an optimum classifier, which are an A and F1 greater than 0.80 and a G-index lower than 0.25. In general, algorithms based on the k-nearest neighbors were found to be associated with greater performance, regardless of the type of feature dataset. In addition, it can also be highlighted that no single classifier of the classifiers trained with the dataset consisting in only baropodometric or mobility analyses achieved sufficient values in all three parameters to be considered an optimum classifier. Just focusing on the MB dataset, all of the classifiers were able to overcome the optimal threshold for the A and F1-score; however, only three classifiers fell into the optimum range of G-index, which are the three types of kNN.

## 4. Discussion

This paper proposes an analysis of the possibility of using machine-learning algorithms to support the clinical assessment of temporomandibular disorders. Within this aim, a comparison among nine machine-learning algorithms, each one trained with three datasets consisting in features gathered from baropodometric and mobility analyses performed with a low-cost and portable sensor system.

By analyzing the general results shown in Figure 8 and Figure 9 and Table 6, qualitative differences associated with the two examined groups can be seen, both concerning the baropodometric and mobility analyses. These findings suggest the potential use of such parameters as inputs for machine-learning algorithms since it is widely acknowledged that machine-learning algorithms exhibit greater performance when working with classes characterized by different motor patterns [21].

Focusing on baropodometric analysis, our results seem to highlight that the presence of TMD can lead to a different distribution of plantar pressure. As a general comment, we can speculate that the physiology impacting the mechanical properties of the temporomandibular joint could also manifest in the feet since they are constantly subjected to cyclic loading [39]. In addition, as also reported in other studies [40,41], a malocclusion may lead to early changes in plantar pressure to be intended as a compensatory mechanism. Nevertheless, it is worth noting that a relationship between TMD and plantar pressure is still questioned, as reported previously, where the absence of correlations has been found [42]. Finally, it must be said that the findings reported here are in contrast to other studies; among others, Souza et al. [14] affirmed how a greater backfoot distribution is typically associated with TMD. It should be underlined that our study does not include any analysis of possible comorbidities, such as, for example, an abnormal body posture; thus, it is not possible to understand if the different foot pressure distribution is due to other factors with respect to TMD.

Similarly, the findings of the cervical mobility are in line with findings already published in the literature, where it is affirmed that the upper cervical range of motion is impaired in patients with temporomandibular disorders [12]. In general, it can be discussed that the patients with TMD are more likely to experience significant cervical hypomobility, since there is a well-known correlation between pain and motor limitations [43]. The findings of lower mobility may be clarified by the convergence of the noxious stimuli from the neck and masticatory structure into the same neuroanatomical structure, known as the trigeminocervical complex [44]. Consequently, experiencing pain in the masticatory system might influence the mobility and strength of the upper neck through mechanisms of somatic referred pain [45]. In addition, the greater reduction in mobility observed for the rotation is confirmed in [46].

Such differences between the two groups are confirmed by the good results achieved by the tested classifiers, especially when considering the ones trained with all of the available features, i.e., the MB dataset. By analyzing the results of the performance indices, it can be affirmed that the selection of the model parameters, which are the kernel function for the SVM, the distance equation for kNN and the number of splits in the DT, seems to be a crucial step only when considering the baropodometric and mobility features separately, whereas no effects are observed when using the MB dataset. Such a result is in contrast with the ones reported in previous studies, where the tuning of model parameters is considered fundamental in all applications [47]. After the selection of the model parameters, by considering all performance indicators, we can affirm that no classifiers met all of the optimal criteria when considering the B dataset, whereas only the c-kNN met the criteria in the case of the M dataset. Conversely, all of the tested classifiers achieved the optimal performance in the case of the MB dataset, with the kNN computed using the cosine distance revealing itself as the best performing. In addition to the best performance in terms of accuracy, F1-score and G-index, kNN could be preferred to support vector machine-based algorithms due to the following: (i) easy implementation since the kNN is a straightforward algorithm; (ii) greater results interpretability since it is easy to understand how the algorithm arrive at its decision; and (iii) few mandatory operations for the training and parameter tuning [48]. Furthermore, a geometric classifier, like the c-kNN, is generally more robust to inter-subject variability than decision trees [49].

In summary, the method proposed here shows promise for integration into the clinical assessment of temporomandibular disorders due to its simplicity and cost-effectiveness. Indeed, it requires only basic motor tasks, known to correlate with TMDs, and utilizes low-cost sensors, which can also be portable to foster the applicability in telemedicine. By employing this protocol as a screening tool, tailored interventions can be devised for patients at an early onset of the pathology. Clinicians are encouraged to incorporate this validated protocol and data analysis methodology into clinical assessment since it has been demonstrated that the early diagnosis and management of TMD can greatly improve prognosis and the quality of life for patients [50]. Moreover, this approach can also assess the effectiveness of customized interventions during the monitoring of the pathology to establish the most efficient care pathways. However, it is important to note that the results obtained in this study are specific to the tested cohort. Further research is needed to determine if this methodology can be applied to diverse demographic groups, including variations in age and comorbidities.

### Limitations

For the sake of completeness, this study reports some limitations. Firstly, we did not perform any analysis to understand the severity level of the TMD; thus, the application of machine-learning algorithms is only based on a binary decision, which is presence or absence of TMD. As a consequence, the proposed methodology cannot substitute the application of more complex procedures, such as the Wilkes classification, but can be exploited as a first investigative approach to avoid useless high-cost and invasive analysis, like radiography. In addition, the number of subjects used to train and test the model does not allow a generalization of the results. In particular, longitudinal tests and the addition of more subject factors can help to elucidate the relationship between an instrument-based approach and clinical scales. Future studies will aim to solve these limitations for a wider perspective on this innovative low-cost methodology for the investigation of TMD.

## 5. Conclusions

Aiming to understand if it is possible to use artificial intelligence for supporting the diagnosis of temporomandibular disorders, we compared nine machine-learning algorithms trained with data related to baropodometric analysis and cervical mobility. Data were gathered from 50 adult participants. The results reveal participants affected by TMDs showed a lower cervical mobility, as well as a different behavior in weight distribution, by preferring to unload the weight on the rearfoot compared with the control group. Such differences were exploited by the machine-learning algorithms, with the one based on k-nearest neighbors with cosine distance found as the best performing in terms of accuracy, F1-score and goodness index. These findings allow us to affirm the potential of artificial intelligence in providing useful information for the diagnosis of TMDs. Thus, the proposed methodology should be considered in the clinical assessment of the pathology, also considering the portability and low-cost of the sensor systems used.

## Figures and Tables

**Table 1:** Mouth opening classification.

Classification		No	Yes
1	Straight (S)	0	1
2	Lateral deviation to the right (RDEV) or to the left (LDEV)	0	1
3	Corrected deviation (CDEV)	0	1
4	Other (O)	0	1

**Table 2:** Joint noises classification.

Classification	Value
No noise	0
Click	1
Crepitus	2

**Table 3:** Muscle palpation and joint palpation classification.

Classification	Pain	
	No	Yes
Temporal Muscle	0	1
Masseter Muscle	0	1
Temporomandibular Joint	0	1

**Table 4:** PHQ-9 questionnaire.

Over the Last 2 Weeks, on How Many Days Have You Been Bothered by Any of the Following Problems?		Not at All	Several Days	More Than Half the Days	Nearly Every Day
1	Little interest or pleasure in doing things	0	1	2	3
2	Feeling down, depressed or hopeless	0	1	2	3
3	Trouble falling or staying asleep, or sleeping too much	0	1	2	3
4	Feeling tired or having little energy	0	1	2	3
5	Poor appetite or overeating	0	1	2	3
6	Feeling bad about yourself–or that you are a failure or have let yourself or your family down	0	1	2	3
7	Trouble concentrating on things, such as reading the newspaper or watching television	0	1	2	3
8	Moving or speaking so slowly that other people could have noticed, or the opposite–being so fidgety or restless that you have been moving around a lot more than usual	0	1	2	3
9	Thoughts that you would be better off dead or of hurting yourself in some way	0	1	2	3

**Table 5:** GAD-7 questionnaire.

Over the Last 2 Weeks, on How Many Days Have You Been Bothered by Any of the Following Problems?		Not at All	Several Days	More Than Half the Days	Nearly Every Day
1	Feeling nervous, anxious or on edge	0	1	2	3
2	Not being able to stop or control worrying	0	1	2	3
3	Worring too much about different things	0	1	2	3
4	Trouble relaxing	0	1	2	3
5	Being so restless it is hard to sit still	0	1	2	3
6	Becoming easily annoyed or irritable	0	1	2	3
7	Feeling afraid as if something awful might happen	0	1	2	3

**Table 6:** Values of the scores associated with the clinical assessments for CG (control group) and TMD (temporomandibular disorders) group, respectively.

Parameters	Related Task	CG	TMD
% of participants manifesting deviation	MO	4%	100%
Mean value (SD) (mm)	OW	43.4 (2.8)	35.4 (2.9)
% of participants manifesting noise	JN	8%	100%
% of participants manifesting muscle pain	MP	40%	100%
% of participants manifesting joint pain	JP	0%	100%
Median score	GAD-7	6	9
Median score	PHQ-9	3	8

**Table 7:** Mean (standard deviation) of the computed features for the two tested groups, CG (control group) and TMD (temporomandibular disorders) group, respectively.

Task		Features	CG	TMD
**Baropodometric** **analysis**	Right foot	PA (°)	7.5 (1.0)	6.0 (0.8)
		FL (%)	19.8 (1.2)	28.5 (1.1)
		RL (%)	31.0 (2.2)	26.2 (1.0)
		TL (%)	52.0 (1.2)	54.4 (1.3)
	Left foot	PA (°)	5.4 (0.8)	4.4 (1.1)
		FL (%)	19.8 (0.7)	24.6 (1.1)
		RL (%)	29.4 (1.4)	21.4 (1.2)
		TL (%)	48.0 (1.1)	45.6 (1.2)
**Cervical mobility**		*θ_FLEX_* (°)	55.9 (4.2)	53.8 (2.2)
		*θ_EXT_* (°)	60.9 (2.9)	52.2 (4.4)
		*θ_FLEX+EXT_* (°)	116.8 (4.4)	106.0 (3.6)
		*θ_R_ROT_* (°)	72.5 (4.3)	67.1 (2.6)
		*θ_L_ROT_* (°)	75.3 (3.9)	70.7 (3.3)
		*θ_TOT_ROT_* (°)	147.7 (4.4)	137.8 (2.2)
		*θ_R_INC_* (°)	43.4 (3.2)	40.5 (3.3)
		*θ_L_INC_* (°)	40.1 (2.3)	40.4 (4.9)
		*θ_TOT_INC_* (°)	83.5 (3.3)	80.5 (3.3)

**Table 8:** Accuracy (*A*), F1-score (F1) and goodness index (*G*) achieved by the nine tested classifiers in the three datasets, where B stands for features related to only baropodometric analysis, M stands for only mobility parameters and MB refers to all of the features used together.

Classifiers	B			M			MB		
	*A*	F1	*G*	*A*	F1	*G*	*A*	F1	*G*
**l-SVM**	0.65	0.76	0.29	0.75	0.77	0.27	0.84	0.80	0.23
**q-SVM**	0.77	0.77	0.31	0.78	0.81	0.26	0.83	0.84	0.22
**c-SVM**	0.69	0.71	0.30	0.79	0.82	0.26	0.80	0.84	0.23
**f-kNN**	0.73	0.79	0.29	0.79	0.81	0.28	0.91	0.93	0.11
**c-kNN**	0.74	0.72	0.33	0.82	0.83	0.22	0.94	0.94	0.08
**w-kNN**	0.72	0.71	0.32	0.75	0.77	0.33	0.93	0.92	0.10
**c-DT**	0.66	0.69	0.45	0.71	0.72	0.30	0.84	0.80	0.24
**m-DT**	0.65	0.65	0.44	0.74	0.75	0.32	0.82	0.83	0.23
**cx-DT**	0.70	0.77	0.34	0.79	0.80	0.26	0.86	0.82	0.23

## Data Availability

For data availability, please refer to the corresponding authors.
